# Prevalence and associated factors of moderate to severe erectile dysfunction among adult men in Malaysia

**DOI:** 10.1038/s41598-023-48778-y

**Published:** 2023-12-06

**Authors:** Muhammad Solihin Rezali, Mohamad Fuad Mohamad Anuar, Mohamad Aznuddin Abd Razak, Zhuo Lin Chong, Azli Baharudin Shaharudin, Mohd Shaiful Azlan Kassim, Mohamed Ashraf Mohamed Daud, Shaiful Bahari Ismail, Zakiah Mohd Said

**Affiliations:** 1 Institute for Public Health, National Institutes of Health, Ministry of Health Malaysia, No.1, Jalan Setia Murni U13/52, Seksyen U13, Setia Alam, 40170 Shah Alam, Selangor Malaysia; 2 Biostatistic and Repository Data, National Institutes of Health, Ministry of Health Malaysia, Shah Alam, Malaysia; 3 Department of Surgery, Hospital USM, Universiti Sains Malaysia, Gelugor, Malaysia; 4 Department of Family Medicine, School of Medical Sciences, Universiti Sains Malaysia, Gelugor, Malaysia; 5 Adult Health Sector, Family Health Development Division, Ministry of Health Malaysia, Shah Alam, Malaysia

**Keywords:** Erectile dysfunction, Epidemiology, Population screening

## Abstract

Erectile dysfunction (ED) is a pervasive problem among men, often shrouded in silence and stigma. This manuscript analysed the National Health and Morbidity Survey 2019 data to identify the prevalence of moderate to severe ED among men aged 18 and above in Malaysia and describe its associated factors. Self-administered questionnaire on ED utilised a locally validated International Index of Erectile Function. Variables on sociodemographics, risky lifestyles and comorbidities were obtained via an interviewer-administered questionnaire. The prevalence was determined using complex sampling analysis, and logistic regression was used to determine the associated factors of ED. A sample of 2403 men aged ≥ 18 participated, with a moderate to severe ED prevalence was 31.6% (95% CI 28.8, 34.6). The mean (± SD) of the total score of IIEF-5 for overall respondents was 18.16 (± 4.13). Multiple logistic regression analysis revealed a significant association between moderate to severe ED among men aged 60 years and above, single or divorcee, men without formal, primary, and secondary education, non-government employees, unemployed, and retiree, as well as physically inactive men. Focused public health interventions are necessary to improve education in sexual health, increase health promotion programs, and promote healthy ageing across the population.

## Introduction

The World Health Organization defines sexual health as “fundamental to the physical or emotional health and well-being of individuals, couples, and families and their social or economic development^1^.” Erectile dysfunction (ED) is a common problem among men worldwide and is one of the burdens of sexual health-related issues. ED is defined as “the consistent or recurrent inability to attain and maintain a penile erection sufficient for sexual intercourse^2^.” The prevalence of ED is expected to increase globally, with estimated range of 3–76.5%^3^. By 2025, 322 million men are expected to be affected by ED worldwide, up from 152 million men in 1995^4^.

Penile erection is a physiological response of neurovascular events integrated with an endocrine and psychological process. It involves smooth muscle relaxation, sinusoidal engorgement with arterial blood, and venous outflow occlusion. Disruption of any of these processes leads to erectile problems. Psychogenic, organic (i.e., neurogenic, hormonal, arterial, cavernosal, or drug-induced), or mixed psychogenic and organic are the three types of ED^5^. The latter was the common type observed in a patient with ED.

The importance of ED as a public health issue has grown exponentially. The prevalence of ED was high among men with underlying medical problems and risky lifestyles, including cardiovascular disease (CVD), hypertension, dyslipidemia, obesity, and smoking^6^. ED itself costs a financial burden to the healthcare system, let alone its complication^7^. A prescribing pattern and cost analysis study in England reported that the rate of primary care prescriptions increased two-fold between 2009 and 2019, owing primarily to more men being screened or seeking ED help^8^. ED shares similar underlying pathophysiology with CVD and mounting evidence that ED substantially raises CVD risk^9^. Early detection of CVD for men with ED as a secondary prevention technique was cost-effective for the healthcare system^10^. Additionally, ED had a detrimental effect on patients' psychosocial well-being and the quality of life of their couples^11^.

Robust epidemiological data clarifying sociodemographic, health-related correlates, and risky lifestyle are undoubtedly essential for a comprehensive understanding of ED. When the modifiable risk factors are identified, effective service delivery, resource allocation and preventive strategies could be established. Therefore, the ED module was included in the National Health and Morbidity Survey (NHMS) 2019: Non-Communicable Diseases. This study aimed to establish the prevalence of moderate to severe ED in Malaysian men aged 18 and above and its associated factors.

## Methods

### Sampling design and sample size

This study was part of the National Health and Morbidity Survey (NHMS) 2019: Non-Communicable Diseases, a nationwide community-based survey conducted in Malaysia in 2019. The NHMS implemented as a cross-sectional study with a two-stage stratified random sampling design to ensure nationally representative data^12^. The primary stratum was in all 13 states and three federal territories in Malaysia, while the secondary stratum was in the locality, urban or rural, within the states. Sample selection consists of two stages, by which the primary sampling unit (PSU) for enumeration blocks (EBs) selection and the secondary sampling unit (SSU) for living quarters (LQs) within the EBs. In this survey, a total of 475 EBs were randomly selected, with 362 EBs from urban areas and 113 EBs from rural areas. Twelve LQs were chosen randomly within the selected EBs. All individuals residing for at least two weeks in the LQs before data collection were eligible to participate in this survey.

For the ED module, the sample size was calculated based on a 6.4% estimated prevalence of ED^13^, design effect of 2.0, and precision of 0.025, which resulted in 2,266 optimum sample sizes. The survey's methodology and sampling design is described in detail in the NHMS 2019 official report^12^. The selection of eligible respondents for the ED module was based on the screening question ‘Are you 18 years and above and sexually active?’ If the response is yes, self-administered ED module would be given to the respondents.

### Measures

#### Sociodemographic characteristics

Sociodemographic characteristics (age, residing location, ethnicity, marital status, education, occupation, household income, and household income category) were collected using a structured questionnaire, similar with the previous NHMS ^12^. The Malaysian government had divided household income into three categories, namely Top 20% (T20), with a monthly income above RM10,970; Middle 40% (M40) with income ranging from RM4851 to RM10970; and lastly Bottom 40% (B40) with earnings of RM4,850 a month or less^14^.

#### ED risk factors

ED risk factors include diabetes mellitus, hypertension, hypercholesterolemia, physical activity, body weight status, smoking and alcohol habits. Non-communicable diseases (diabetes mellitus, hypertension, or hypercholesterolemia) variables were defined as respondents who reported having these illnesses (self-reported) and raised blood glucose, blood pressure, or cholesterol during clinical assessments amongst those not known to have these conditions. Clinical assessments and biochemical tests were conducted by trained nurses for fasting or random blood glucose, cholesterol level, blood pressure and anthropometric measurements.

#### ED

Self-administered questionnaire on validated 5-item English and Malay versions of the International Index of Erectile Function (IIEF-5) was distributed to the eligible respondents (sexually active males 18 years old and above). Each item in the IIEF-5 assesses a different domain of erectile function, i.e., erection confidence, erection firmness, maintenance frequency, maintenance ability, and intercourse satisfaction. For each item, respondents could assign a score from 1 to 5, with a higher score indicating better function. Total scores on this scale ranged from five to 25, and is classified as normal (score 22–25), mild ED (score 17–21), moderate ED (score 8–16), and severe ED (score 5–7)^15^. Since both moderate and severe ED are more likely to require clinical treatment than mild ED, they were combined and highlighted for this paper^16,17^.

### Data collection

A training workshop for field supervisors, data collectors, and nurses was conducted prior to the data collection. The primary objectives of the training were to familiarize the data collection teams with the questionnaires, to develop interpersonal skills, and to appreciate the need for good teamwork. Data collection was initiated from July 2019 to October 2019, covered all states and federal territories in Malaysia. Data were sent to the Institute for Public Health for quality control and database management.

### Statistical analysis

Data analysis was calculated using a complex sample module in IBM Statistical Package for Social Sciences (SPSS) for Windows version 21.0 (IBM Corp., Armonk, NY, USA). A weighting factor was applied, considering the design weight and non-response, and post-stratification adjustment was done for age, sex, and ethnicity. The detailed calculation for the weighting factor was stated in the NHMS 2019 report^12^. Complex sample analysis was done to illustrate the mean of IIEF-5 score within the respective domains and determine the prevalence of moderate to severe ED by sociodemographic characteristics. Factors associated with moderate to severe ED were determined at both univariate and multivariable levels by using simple logistic regression and multiple logistic regression, respectively. The outcome was a binary variable coded as “0” for normal and mild ED and “1” for moderate to severe ED. Variable selection was made using the backward stepwise logistic regression method. From the simple logistic regression analysis, factors with *p* value < 0.25 were included for further analysis. The final model was presented with adjusted odd ratio (AOR), beta coefficient (b), and *P* value, with a level of significance at *P* value less than 0.05. Multicollinearity and two-way interaction term were checked. Hosmer–Lemeshow test, classification table, and the receiver operating characteristic (ROC) curve were done to check for model fitness.

### Ethics approval

All respondents were given a bilingual (Malay and English) consent form that detailed the survey's purpose and methodology. All procedures were approved and granted ethical approval by the Medical Research and Ethics Committee of the Ministry of Health Malaysia. This study was registered with the National Medical Research Register (NMRR) as NMRR-18-3085-44207. The survey was conducted according to the Declaration of Helsinki and the Ministry of Health Malaysia guidelines and regulations to ensure that the ethical was abide during the data collection.

## Results

**Figure 1:**
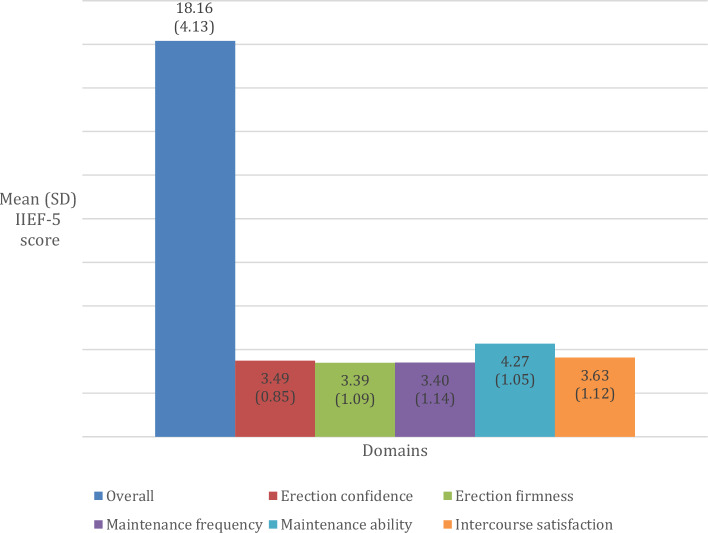
Descriptive mean score of IIEF-5.

A total of 3,207 adult males were eligible for the ED module, and 2403 of them completed all the questionnaires provided, making the response rate of this study 73.5%. Table 1 shows the sociodemographic characteristics of sexually active men aged 18 and above. Figure 1 shows the mean scores according to IIEF-5 domains. Overall, the total mean score of the IIEF-5 was 18.16 ± 4.13. By domains, erection firmness shows the lowest mean score, followed by maintenance frequency and erection confidence (Fig. 1).

**Table 1:** Socio-demographic characteristics of the sexually active men aged 18 years old and above (n = 2403).

Sociodemographic characteristics	Count	Percentage (%)
Location		
Urban	1541	80.2
Rural	862	19.8
Age group (Years)		
18–30	381	25.6
31–59	1618	64.7
60 and above	404	9.7
Ethnicity		
Malay	1655	53.6
Chinese	246	19.5
Indian	143	5.7
Bumiputera Sabah & Sarawak	247	11.3
Others	112	9.9
Marital status		
Single/Divorcee	299	16.6
Married	2104	83.4
Education		
No formal education	45	2.1
Primary education	403	16.6
Secondary education	1292	51.5
Tertiary education	662	29.7
Occupation		
Government employee	396	11.3
Private employee	967	49.2
Self employed	639	25.9
Not working/unpaid worker/homemaker/student	210	8.4
Retiree	191	5.2
Household income (RM)		
Less than RM1000	161	5.6
RM 1000–RM 3999	1150	51.0
RM 4000–RM 7999	678	28.4
RM 8000 and above	360	15.0
Household income category		
Bottom 40% (B40)	1489	63.3
Middle 40% (M40)	608	26.1
Top 20% (T20)	252	10.6

Our study revealed that 31.6% of sexually active men 18 years and above reported having moderate to severe ED. The moderate to severe ED prevalence was high among rural dwellers, men aged 60 years and above, single or divorcees, people with primary education, unemployment, retiree, and people in the lowest household income category (Table 2).

**Table 2:** Prevalence of moderate to severe erectile dysfunction among sexually active men aged 18 years old and above by sociodemographic and ED risk factors (n = 2403).

Sociodemographic characteristics	Unweighted count	Estimated population	Moderate-severe ED		
			Prevalence (%)	95% CI	
				Lower	Upper
Overall	821	1,744,121	31.6	28.8	34.6
Location					
Urban	484	1,356,839	30.6	27.3	34.2
Rural	337	387,282	35.5	31.1	40.2
Age Group (Years)					
18–30	138	458,046	32.7	25.6	40.6
31–59	432	941,710	26.3	23.2	29.6
60 and above	251	344,365	64.1	57.3	70.4
Ethnicity					
Malay	541	914,478	30.9	28.0	33.9
Chinese	92	336,753	31.3	23.8	40.0
Indian	54	107,843	34.6	25.4	45.0
Bumiputera Sabah & Sarawak	94	243,338	39.2	32.9	46.0
Others	40	141,709	26.0	15.6	40.0
Marital status					
Single/Divorcee	156	482,873	52.7	44.2	61.1
Married	665	1,261,247	27.4	24.6	30.4
Education					
No formal education	22	40,300	38.6*	17.8	64.5
Primary education	202	358,173	39.1	30.6	48.4
Secondary education	446	1,009,628	35.5	32.0	39.3
Tertiary education	145	322,249	19.7	15.4	24.7
Occupation					
Government employee	66	105,047	16.9	12.2	22.9
Private employee	281	787,299	29.0	25.1	33.3
Self employed	251	453,211	31.7	25.8	38.2
Not working/unpaid worker/homemaker/student	120	249,714	53.9	42.6	64.8
Retiree	103	148,850	51.9	42.1	61.5
Household income (MYR)**					
Less than 1000	61	105,398	56.3	43.2	68.7
1000–3999	457	938,855	32.0	27.8	36.5
4000–7999	192	456,881	29.9	24.8	35.4
8000 and above	82	193,498	23.9	18.6	30.1
Household income category					
Bottom 40% (B40)	546	109,0600	33.1	29.2	37.3
Middle 40% (M40)	190	463,661	30.5	25.2	36.3
Top 20% (T20)	56	140,372	24.6	18.3	32.1
ED risk factors					
Diabetes mellitus	181	308,822	28.7	23.7	34.4
Hypertension	276	560,662	30.7	26.1	35.6
Hypercholesterolemia	321	670,298	34.6	30.5	38.9
Physical inactivity	196	400,019	40.9	34.6	47.6
Abdominal obesity	388	724,236	30.1	26.4	34.0
Current smoker	313	676,410	28.4	24.3	32.9
Past smoker	87	189,518	31.1	23.3	40.2
Smokeless tobacco	81	216,986	33.0	23.3	44.4
E-cigarettes/Vape	60	163,195	31.3	20.3	45.0
Ever drinker	122	359,717	29.6	23.3	36.7
Current drinker	100	325,045	31.0	23.9	39.0
Binge drinker	56	168,567	30.6	20.6	42.9

*Prevalence with high relative standard error (RSE), interpret with caution.

**MYR1.00 = USD0.21 on 6 October 2023.

The association between moderate to severe ED and sociodemographic factors, medical conditions, and other risk factors were summarised in Table 3. Men aged 60 years and above were strongly associated with moderate to severe ED with AOR of 3.04 (95% CI 2.27, 4.10). Single or divorcees also showed higher odds with AOR of 2.88 (95% CI 2.10, 3.96) than married men. By educational status, no formal education, primary and secondary education were significantly associated with moderate to severe ED with AOR 3.04 (95% CI 1.52, 6.05), 2.30(95% CI 1.69, 3.12) and 1.81(95% CI 1.43, 2.28), respectively. Private employees, self-employed, those who were not working, unpaid workers, homemakers, students, or retirees were also associated with moderate to severe ED. Finally, physically inactive men were significantly associated with moderate to severe ED with AOR of 1.50 (95% CI 1.19, 1.89).

**Table 3:** Association of Sociodemographic and Risk factors towards moderate to severe Erectile Dysfunction (ED) status using Logistic Regression (n = 2403).

Factor	Simple Logistic Regression (SLR)			Multiple Logistic regression (MLR)*		
	b	Crude OR (95% CI)	*p* value	b	Adjusted OR* (95% CI)	*p* value
Location						
Urban	–	1	–	–	1	–
Rural	0.338	1.402 (1.178, 1.668)	< 0.001	− 0.008	0.992 (0.804, 1.225)	0.943
Age group (Years)						
18–30	0.459	1.582 (1.249, 2.003)	< 0.001	− 0.085	0.919 (0.678, 1.246)	0.585
31–59	–	1	–	–	1	–
60 and above	1.508	4.518 (3.592, 5.682)	< 0.001	**1.114**	**3.045 (2.267, 4.091)**	** < 0.001**
Ethnicity						
Malay	–	1	–	–	1	–
Chinese	0.213	1.237 (0.937, 1.633)	0.134	0.041	1.042 (0.745, 1.456)	0.812
Indian	0.228	1.256 (0.882, 1.789)	0.206	0.301	1.351 (0.907, 2.013)	0.139
Bumiputera Sabah & Sarawak	0.275	1.316 (0.999, 1.734)	0.051	0.225	1.253 (0.909, 1.726)	0.169
Others	0.140	1.150 (0.771, 1.716)	0.493	0.192	1.212 (0.770, 1.908)	0.406
Marital status						
Single/divorcee	0.859	2.361 (1.848, 3.015)	< 0.001	**1.058**	**2.881 (2.099, 3.955)**	** < 0.001**
Married	–	1	–	–	1	–
Education						
No formal education	1.677	5.348 (2.865, 9.984)	< 0.001	**1.111**	**3.038 (1.526, 6.049)**	**0.002**
Primary education	1.276	3.583 (2.740, 4.687)	< 0.001	**0.833**	**2.301 (1.696, 3.121)**	** < 0.001**
Secondary education	0.631	1.880 (1.513, 2.335)	< 0.001	**0.592**	**1.808 (1.432, 2.283)**	** < 0.001**
Tertiary education	–	1	–	–	1	–
Occupation						
Government employee	–	1	–	–	1	–
Private employee	0.717	2.048 (1.520, 2.761)	< 0.001	**0.440**	**1.553 (1.134, 2.126)**	** < 0.001**
Self employed	1.174	3.235 (2.376, 4.403)	< 0.001	**0.760**	**2.138 (1.539, 2.970)**	** < 0.001**
Not working/unpaid worker/homemaker/student	1.897	6.667 (4.558, 9.750)	< 0.001	**0.811**	**2.250 (1.462, 3.463)**	** < 0.001**
Retiree	1.767	5.852 (3.969, 8.629)	< 0.001	**0.796**	**2.216 (1.405, 3.495)**	**0.001**
Household income (MYR)**						
Less than 1000	1.308	3.698 (2.491, 5.492)	< 0.001	0.523	1.668 (0.881, 3.235)	0.115
1000–3999	0.720	2.055 (1.563, 2.702)	< 0.001	0.198	1.219 (0.712, 2.087)	0.471
4000–7999	0.292	1.339 (0.995, 1.804)	0.054	− 0.003	0.997 (0.634, 1.568)	0.990
8000 and above	–	1	–	–	1	–
Household income category						
B40	0.627	1.872 (1.373, 2.553)	< 0.001	0.190	1.209 (0.747, 1.956)	0.440
M40	0.404	1.498 (1.068, 2.101)	0.019	− 0.060	0.942 (0.536, 1.657)	0.835
T20	–	1	–	–	1	–
Diabetes mellitus						
No	–	1	–	–	1	–
Yes	− 0.236	0.790 (0.647, 0.964)	0.021	− 0.119	0.888 (0.702, 1.123)	0.321
Hypertension						
No	–	1	–	–	1	–
Yes	− 0.334	0.716 (0.600, 0.854)	< 0.001	− 0.151	0.860 (0.693, 1.068)	0.173
Hypercholesterolemia						
No	–	1	–	–	1	–
Yes	− 0.117	0.890 (0.749, 1.057)	0.184	0.000	1.000 (0.812, 1.231)	0.998
Physical activity						
Active	–	1	–	–	1	–
Inactive	0.440	1.552 (1.261, 1.912)	< 0.001	**0.410**	**1.506 (1.196, 1.897)**	** < 0.001**
Body mass index (BMI)						
< 25.00	–	1	–	–	1	-
25.00 and above	− 0.189	0.828 (0.697, 0.983)	0.031	0.017	1.017 (0.838, 1.235)	0.861
Abdominal obesity						
No	–	1	–	–	1	–
Yes	− 0.025	0.975 (0.821, 1.158)	0.773	–	−	–
Current smoker						
No	–	1	–	–	1	–
Yes	0.016	1.016 (0.857, 1.203)	0.857	–	–	–
Past smoker						
No	–	1	–	–	1	–
Yes	0.076	1.079 (0.809, 1.440)	0.604	–	−	–
Smokeless tobacco						
No	–	1	–	–	1	–
Yes	− 0.106	0.899 (0.681, 1.188)	0.455	–	–	–
E-cigarettes/Vape						
No	–	1	–	–	1	–
Yes	− 0.203	0.816 (0.596, 1.118)	0.257	–	–	–
Ever drinker						
No	–	1	–	–	1	–
Yes	− 0.110	0.896 (0.709, 1.132)	0.356	–	–	–
Current drinker						
No	–	1	–	–	1	–
Yes	− 0.060	0.942 (0.729, 1.217)	0.648	–	–	–
Binge drinker						
No	–	1	–	–	1	–
Yes	0.231	1.260 (0.779, 2.039)	0.346	–	–	–

*Adjusted for all sociodemographic and risk factors for ED, Backward LR Multiple Logistic regression was applied. Multicollinearity and interaction were checked and not found. Hosmer–Lemeshow test: *P* value = 0.578. Classification table: 71.0. ROC Curve: 0.718 (95%CI 0.696, 0.739; *p* value: < 0.001).

**MYR1.00 = USD0.21 on 6 October.

Significant values are in [bold].

## Discussion

From the IIEF-5 descriptive review, the total mean score was 18.16 (SD ± 4.1), comparable to the Turkish study with a mean score of 18.20 (SD ± 6.2)^18^. This was lower than another study done in Vienna which they found the average score was 21.3 (SD ± 4.9)^19^. A longitudinal study by Imai et al. reported that the mean score of ED decreased by year and rapidly declined in the older age group^20^. This may indicate that the prevalence of moderate to severe ED was higher in elderly than younger group. Based on our findings, moderate to severe ED prevalence was 31.6% (CI 28.8, 34.6). According to the Massachusetts Male Aging Study (MMAS), 34.8% of men aged 40–70 had moderate to severe ED^21^. A study done by Nicolosi et al. found that, the age-adjusted prevalence of moderate to severe ED was 17% in Italy, 15% in Brazil and 34% in Japan. Local studies reported that moderate to severe ED prevalence in Malaysia ranged from 40.8 to 46%^22,23^. These differences in prevalence may reflect actual demographic differences and cultural differences in the perceptions and attitudes toward ED, as the aforementioned studies were conducted among urban residents aged 40 years and older.

The high ED prevalence among older age groups was anticipated, as seen in various studies^6,24–26^. However, it was not usually expected in younger age groups. In our findings, the high prevalence of moderate to severe ED among 18–30 years old is worth discussing, even though it is not statistically significant compared to those in the 31–59 years group. The pattern of the exceptionally high prevalence of ED among young men had been observed in various studies, especially those studies using the IIEF as ED measurement tools^26,27^. The comparatively high prevalence of ED among young men may be explained by psychological variables such as inexperience with sexuality, performance anxiety, and life pressures. A cohort study among sexually active young adult men revealed that a history of depression, antidepressants usage, and anxiety leads to higher odds of having moderate to severe ED^28^. A recent study found an increased occurrence of ED in men under the age of 40, and this pattern is likely underrated due to younger populations' under-reporting^29^. Although ED in young adult men was believed to be due to psychogenic factors, there were increased amounts of data regarding ED as a proxy of cardiovascular, diabetes and overall men’s health^30^. A careful and comprehensive general health assessment of patients complaining of ED should be carried out, regardless of patient’s age.

From the multiple logistic regression analysis, men aged 60 years and above were three times more likely to have moderate to severe ED than the 31–59 years group. Advanced age has been considered the main unmodifiable risk factor for ED, with signs and symptoms most typically occurring in men over the age of 65^31,32^. The ageing process can affect all the components in our body (nerves, arteries, veins, muscles, and hormones), including those needed in erection function^29^. ED is often believed to be a regular part of the ageing process. However, this assumption may not be entirely accurate as ED is not just a natural result of ageing where to be accepted alongside other aging-related disorders. For the elderly, ED may occur due to specific illnesses or adverse treatment for certain diseases^31^. Ageing is unavoidable; however, maintaining good health and controlling chronic illness will help to mitigate potential health-related problems.

From our study, single or divorcee men were found to be associated significantly with moderate to severe ED, which was also observed in a number of studies^28,33^. Single respondents, especially teenagers and young adults, may lack experience and knowledge regarding their sexual abilities. A previous study reported that lack of sexual knowledge and anxiety are common contributing factors to ED^29^. A study done in Thailand found that married men rate their sexual abilities better than single, separated, divorced, and widowed males^34^. Unstable relationships, stress, depression, and emotional issues can be related to sexual problems and ED^35^. Based on NHMS 2019, single men and divorcees reported having a higher prevalence of depression than married men^12^. This finding corresponds to our results with the higher prevalence of moderate to severe ED among single and divorcee men than in the married group. Early sexual education and relationship counselling can be beneficial in preventing ED and encourage people to recognize their health, well-being, and dignity while developing respectful social and sexual relationships.

There was an inverse relationship between education and presence of moderate to severe ED. Similar findings were observed from various studies where low educational status was associated with ED^27,35^. The current study clearly showed the odd ratio reduced by education level. This association possibly explains that educated people have more knowledge and self-awareness regarding their sexual abilities and, hence, take preventive measures or treatment^36^. It is also possible that people with a higher education level had better socioeconomic status, thus had better access to healthcare facilities, and could afford better treatment^37^. A worsening economic situation causes more stress, which burdens sexual function. These would explain the high prevalence of ED in those not working and low household income.

In terms of occupation, non-governmental employees were more likely to have moderate to severe ED than government employees. There was no apparent reason for this. The aetiology of these observations is most likely complex. Working conditions, stressors, and lifestyle issues are almost certainly related. Several studies, however, indicated that particular types of occupations were associated with ED. According to the MMAS longitudinal study, men in blue-collar jobs were more likely to acquire ED than men in white-collar jobs^38^. In their review, Burnett et al. discovered a probable risk relationship between environmental exposures and ED. Environmental toxicants have been postulated to have a detrimental effect on erectile function primarily through their effects on the neurological and hormonal systems^39^.

There was a statistical difference in moderate to severe ED by comparing physical activity level. Lack of physical activity and sedentary lifestyles were strongly associated with ED^40^. A study by Cheng et al. concluded that moderate to high levels of physical activity reduced ED risk^41^. ED patients reported that moderate to vigorous-intensity aerobic exercise would enhance their sexual well-being^42^. An active lifestyle is the best way to increase nitric oxide (NO) and testosterone levels, improve body image, and reduce stress and anxiety^43^. A strategic plan is required to enhance physical activity among the population by promoting active commuting among adults and strengthening the knowledge of physical activity in the community. The transformation of sedentary lifestyles and increased physical activity might help to combat the growing epidemics of obesity and age-related diseases such as cardiovascular disorders, chronic illnesses, and ED—an unforeseen positive side effect of regular physical activity adopting in our lives.

Our study discovered that no significant association between moderate to severe ED and chronic disease (Diabetes, Hypertension, and Hypercholesterolemia) or risky behaviour (obesity, smoking, and alcohol consumption). These patterns were also observed in a few studies among the younger population^28,44^. One possible reason is that the study's youthful population suffered from these modifiable risk factors but had not yet experienced the consequences of these problems, which all have a detrimental effect on the vascular system over time^45^.

These findings suggest that ED issues in Malaysia need immediate attention and collaboration with multidisciplinary teams to handle this issue. The introduction of a formal sexual reproductive health education via a comprehensive syllabus or programs should begin from a younger age and be suited for multiethnicity and multicultural countries like Malaysia. Furthermore, advocate on expanding health promotion services, significantly to halt the progress of NCD and risky lifestyle through talks, campaigns, and social media promotion. In addition, a collaboration with multiagency is vital to raise awareness of sexual reproductive health among the community. Finally, healthcare workers should empower themselves with knowledge and training programs on diagnosing and managing ED. A future research design may be necessary to close the knowledge gap in ED in various community settings.

The study's main strength was the nationally representative sample, which allows the results to be applied to the entire Malaysian population. The large sample size ensures sufficient statistical capacity when estimating the prevalence and its associated factors. Furthermore, the validity of our self-reported data was assured by the use of structured questionnaires, self-administered data collection, and firm quality control during the survey duration. However, a few drawbacks to this research should be listed. Firstly, the cross-sectional study design eliminates the probability of a causal relationship between the associated factors and ED. Secondly, a wide range of age categories, as well as the fact that the results were focused on self-perceptions rather than clinical evaluations, could present information bias such as recall bias and misreporting that might obscure the actual issues.

## Conclusion

In conclusion, ED is prevalent in Malaysia, with 31.6% of sexually active men aged 18 years and above complaining of moderate to severe ED. Age 60 years and above, single or divorcee, low educational level, non-governmental employees, and physically inactive men were significantly associated with moderate to severe ED. Therefore, focused public health interventions are necessary to improve education in sexual health, increase health promotion programs, and promote healthy ageing across the population.

## Data Availability

The data used to support the findings of this study are available from the corresponding author upon reasonable request.
